# Widespread and Long-Enduring Hyperkeratosis Lenticularis Perstans (Flegel’s Disease): Clinico-Pathological and Dermoscopic Features of a Rare Presentation

**DOI:** 10.3390/dermatopathology10010006

**Published:** 2023-01-13

**Authors:** Giorgio Stabile, Giovanni Paolino, Nathalie Rizzo, Franco Rongioletti

**Affiliations:** 1 Dermatologia Clinica, Università Vita-Salute San Raffaele, 20132 Milan, Italy; 2 Dermatologia, IRCCS Ospedale San Raffaele, 20132 Milan, Italy; 3 Surgical Pathology, IRCCS Ospedale San Raffaele, 20132 Milan, Italy

**Keywords:** hyperkeratosis lenticularis perstans, Flegel’s disease, histopathology, dermoscopy

## Abstract

Hyperkeratosis lenticularis perstans, also known as Flegel’s disease (FD), is a rare cutaneous disorder affecting mainly the lower extremities of middle-aged people. Due to its rarity, this disease is usually not recognized by physicians resulting in a delay in diagnosis, especially in those cases with atypical cutaneous involvement. Herein, we present a 72-year-old woman who developed FD characterized by a generalized distribution, involving, in addition to the lower limbs, the trunk and the upper limbs as well. We performed a description of the dermoscopic and pathologic features of this rare entity, also carrying out a brief reappraisal of the cases of FD with a diffuse, atypical and generalized distribution that have been described in the literature. Histopathology with clinical correlation is the cornerstone of the diagnosis, even and especially in atypical cases. This patient with a disease duration of 58 years also represents the longest-lasting case of FD reported in the literature.

## 1. Introduction

Hyperkeratosis lenticularis perstans, also known as Flegel’s disease (FD), is a rare benign cutaneous disorder (first described in 1958) presenting with multiple reddish-brown keratotic papules on the lower extremities of mid-to-older age people. Rarely, FD can affect other body sites or present with a diffuse and generalized involvement, making diagnosis more difficult. Herein, we report the case of a woman who developed a generalized form of FD with a disease duration of 58 years, describing its histopathologic and dermoscopic findings. A brief review of the atypical and generalized cases of this rare entity reported in the literature so far are also described.

## 2. Case Report

**Figure 1:**
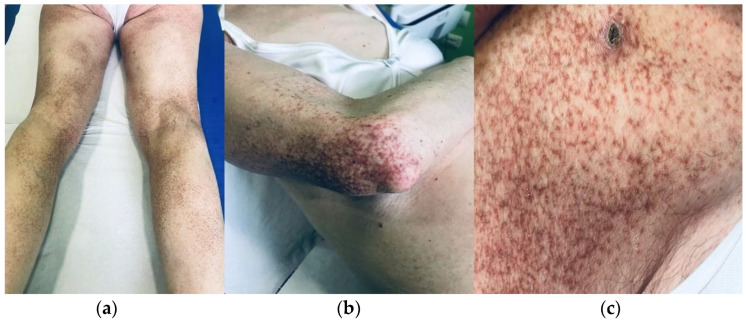
Multiple small, reddish and brownish, hyperkeratotic papules, initially present only in the lower limbs (**a**) and subsequently involving also on upper limbs (**b**) and the trunk (**b**,**c**).

**Figure 2:**
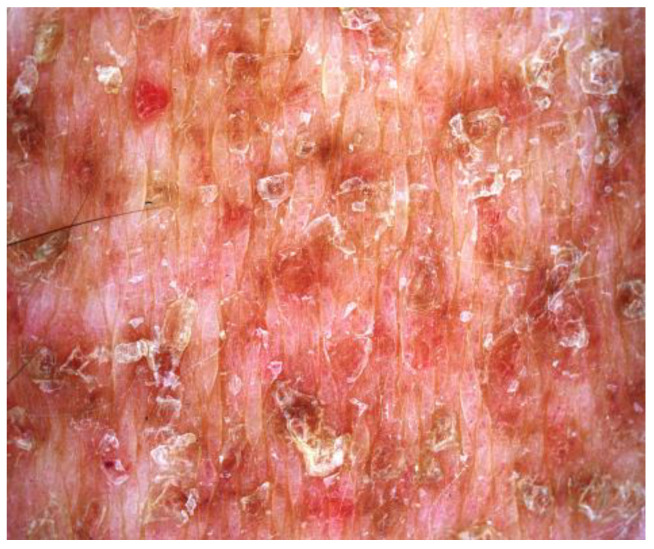
Dermoscopy showed brownish structureless areas, with a brownish pseudo-reticular appearance on an erythematous base, rare ectatic lacunae-like vessels and hyperkeratosis with fine superficial desquamation.

**Figure 3:**
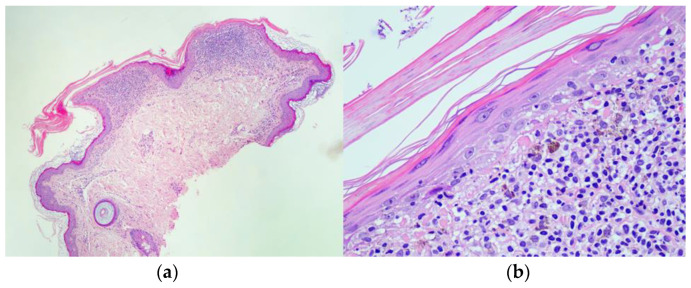
Histopathology showed a dense, lichenoid lymphocytic infiltrate in the superficial dermis, with focal accentuation in the papillomatous areas, hyper and parakeratosis, and thin, atrophic cutaneous epidermis under hyper-parakeratotic scales (**a**; Hematoxylin and Eosin 10×); Close-up of the lesion showing atrophic epidermis under hyper-parakeratotic scales, lichenoid infiltrate in the superficial dermis characterized by the presence of multiple small lymphocytes, with scattered melanophages, exocytosis and basal vacuolization of the epidermis (**b**; Hematoxylin and Eosin 30×).

**Figure 4:**
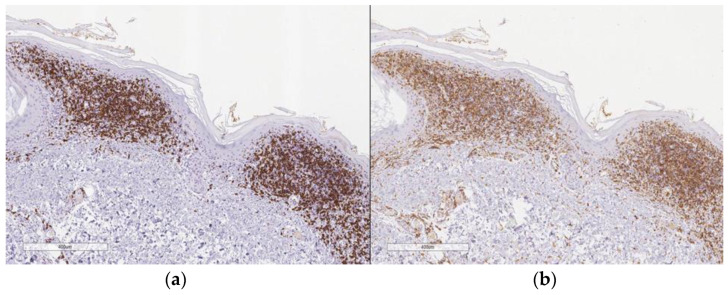
CD8 immunostain (**a**; 10×); CD4 immunostain (**b**; 10×).

A 72-year-old Caucasian woman was seen for a 58 year old history of multiple small, reddish and brownish, keratotic papules, initially located on her lower limbs, which subsequently spread, involving the upper limbs and the trunk (Figure 1a,b). Her personal medical history was negative for cutaneous and systemic diseases, except for an in situ well differentiated cutaneous squamous cell carcinoma on the leg, which was removed two years before. Dermoscopy of one cutaneous lesion showed brownish structureless areas, with an also brownish pseudo-reticular appearance on an erythematous base, some scattered lacunae-like ectatic vessels and hyperkeratosis with fine superficial desquamation (Figure 2). Histopathology of a lesion on the trunk showed a compact, thick lamellar hyperkeratosis with focal parakeratosis overlying an atrophic epidermis associated with a dense, lichenoid infiltrate of small lymphocytes in the superficial dermis with focal accentuation in the papillomatous areas. Some melanophages, focal basal vacuolization with scattered areas of exocytosis, were also present (Figure 3a,b). Immunohistochemical studies showed a similar proportion of CD4 and CD8T-lymphocytes (Figure 4a,b) with sparse to absent CD20 cells. According to the clinico-pathologic correlation, a final diagnosis of generalized hyperkeratosis lenticularis perstans (Flegel’s disease) was made. A therapeutic attempt with acitretin 20 mg/die was made and stopped after 3 months due to scarce improvement and poor satisfaction of the patient, in the absence of side effects.

## 3. Discussion

Flegel’s disease (FD), first described in 1958, is a rare cutaneous disorder that usually arises in the fourth/fifth decade of life, often confused with other cutaneous keratotic pathologies. To the best of our knowledge, about 80 articles dealing with FD cases have been reported in the literature [1,2,3,4,5,6,7,8,9,10,11,12,13,14,15,16,17,18,19,20,21,22,23]. According to the literature, there is a slight prevalence of the female gender with a ratio 1.6:1 and a median age of 50 years, ranging between 18 and 82 years [1,2,3,4,5,6,7,8,9,10,11,12,13,14,15,16,17,18,19,20]. The median duration of the disease before reaching the diagnosis is reasonably long, with a median of eight years. FD is mainly characterized by small and multiple erythematous brownish and hyperkeratotic papules, from 1 mm to 5 mm in diameter, whose typical location is on the dorsa of the feet and lower part of the legs in 87% of cases. An atypical presentation with involvement of unusual sites such as the axillae, eyes, palms/soles, a single leg, trunk, upper extremities, buttocks, forehead, pinnae, breast, hands and oral mucosa has been also reported [1,2,3,4,5,6,7,8,9,10,11]. A diffused or generalized involvement of the body is a very rare presentation with only three cases described [2,5,6]. In Table 1 we summarize all the cases of FD in the literature that showed a generalized distribution, as well as cases with involvement of atypical anatomic sites, highlighting how the duration of the disease in all these cases is long, with a mean time of 16 years before reaching a diagnosis, ranging between 5 years and 30 years. In this regard, the main point of interest in the present case is the longstanding history of the disease, lasting 58 years, with an atypical, widespread and generalized distribution of the lesions involving the lower limbs, the upper limbs and the chest.

Regarding the pathogenesis, since there are familial cases, FD has been hypothesized to be an autosomal dominant disease (although no specific candidate genes have yet been discovered); however, since most cases seem to occur sporadically and usually start late in adult life, the hypothesis of a genetic pathogenesis is not strengthened. It has been suggested that FD is a primary keratinization disorder. In this regard, while some authors have found some changes in the membrane-coating granules (MCGs), inducing structural alterations in the epidermis, other authors have not found these alterations [1,13,14]. A possible correlation of FD with other conditions, such as *Borrelia burgdorferi* [15], basal cell carcinoma, squamous cell carcinoma, urinary bladder tumor, lung cancer and digestive cancers has also been reported [16]. Anecdotal association with hyperaldosteronism and hyperthyroidism, as well as a coexistence with Kyrle disease and Mibelli porokeratosis have been also described [17]. The personal medical history of our patient was negative, except for a cutaneous squamous cell carcinoma on her leg that was removed two years before. Therefore, the correlation between FD and cutaneous keratinocyte malignancies should be better evaluated in future reports.

The main clinico-pathologic differential diagnoses of FD are stucco keratoses, disseminated superficial actinic porokeratosis (DSAP), Kyrle’s disease, acrokeratosis verruciformis of Hopf and porokeratosis of Mibelli [21,22]. In all these cases, the histological examination and the clinico-pathologic correlation help perform a correct diagnosis.

To date, dermoscopy of FD has been performed in only two cases [18,19] presenting with superficial whitish scaling areas (induced by hyper and parakeratosis), brownish structureless areas (induced by melanophages in the dermis, produced by the lichenoid infiltrate and the pigment incontinence) and grayish areas induced by lichenoid infiltrate. In our case, we found similar dermoscopic findings, with some superficial scaling areas, brownish reticulated areas and multiple lacunae-like areas corresponding to superficial dermis ectatic vessels.

The treatment of FD is challenging; the main treatments include emollients, topical steroids (betamethasone diproprionate 0.05%), 5-fluorouracil (5-FU) cream, topical or systemic retinoids, Vitamin D3 and PUVA. Ablative treatments (local excision, CO_2_ laser, curettage, dermabrasion, cryotherapy and electrocoagulation) can also be taken into consideration [1]. However, all these treatments offer only partial therapeutic responses and are aimed only at ameliorating the aesthetic impact of the lesions, rather than at their definitive treatment.

## 4. Conclusions

FD, or hyperkeratosis lenticularis perstans, is a rare disease whose diagnosis is based upon a good clinico-pathological correlation, as there are no genetic or laboratory exams specific to this disease. When an atypical presentation occurs, such as in our case, with a diffuse and generalized involvement, the diagnosis is more difficult and can be delayed. Histopathology with the typical findings of focal, thick, compact hyperkeratosis with parakeratosis, thinned epidermis and a dense band-like lymphocytic inflammatory infiltrate in the papillary dermis is mandatory to confirm the diagnosis. Our patient is also the longest-enduring case of FD, lasting 58 years, with progressive spreading from the legs to a generalized involvement.

## Figures and Tables

**Table 1:** Review of the clinical features of generalized hyperkeratosis lenticularis perstans including involvement of atypical areas, reported so far in the literature [2,3,4,5,6,7,8,9,10,11,20].

Authors	Year	Gender	Age	Duration (Years)	Body Area
Krishnan A et al.	2012	M	25	15	Trunk, limbs, face
Fernandez et al.	2009	NF	NF	NF	Palms and soles
Miranda et al.	1998	F	29	16	Single leg involvement
Jang et al.	1999	M	67	NF	Trunk, upper extremities
Kocsard et al.	1968	M	71	20–30	Trunk, upper extremities
Price et al.	1987	F *	36 *	23 *	Buttocks, forehead, pinnae
Urbina et al.	2016	F	60	5	Breast
Mattaredona et al.	2009	M	60	10	Single leg involvement
Massone et al.	1990	F	55	NF	Hands
van de Staak et al.	1980	M **	62	20 **	Oral mucosa
Present case	2022	F	72	58	Upper limbs, lower limbs, chest
Miljković J	2004	F	82	11	Upper and lower limbs
De La Pinta et al.	2020	F	79	1	Back and breast

M means male; F means female; * Kocsard et al. reported a case series of 12 female patients, among which 11 patients showed atypical anatomic localizations, with a median age of 40 (ranging between 18 and 51) and a median duration of the symptoms of 23 years (ranging between 4 and 43 years); ** van de Staak et al. reported 2 male patients (mean age 62, ranging between 55 and 69) with Flegel’s disease (FD) with abnormalities of the mucosal surface. The information about the duration of the cutaneous manifestations was reported only for one case; NF means not found.

## Data Availability

Not applicable.
